# Beware: Recruitment of Muscle Activity by the EEG-Neurofeedback Trainings of High Frequencies

**DOI:** 10.3389/fnhum.2017.00119

**Published:** 2017-03-20

**Authors:** Katarzyna Paluch, Katarzyna Jurewicz, Jacek Rogala, Rafał Krauz, Marta Szczypińska, Mirosław Mikicin, Andrzej Wróbel, Ewa Kublik

**Affiliations:** ^1^Department of Neurophysiology, Nencki Institute of Experimental Biology of Polish Academy of ScienceWarsaw, Poland; ^2^Centre for Physical Education and Sport, Military University of TechnologyWarsaw, Poland; ^3^Department of Physical Education, University of Physical EducationWarsaw, Poland

**Keywords:** artifacts, attention, beta rhythm, biofeedback, muscle control, placebo

## Abstract

EEG-neurofeedback (NFB) became a very popular method aimed at improving cognitive and behavioral performance. However, the EMG frequency spectrum overlies the higher EEG oscillations and the NFB trainings focusing on these frequencies is hindered by the problem of EMG load in the information fed back to the subjects. In such a complex signal, it is highly probable that the most controllable component will form the basis for operant conditioning. This might cause different effects in the case of various training protocols and therefore needs to be carefully assessed before designing training protocols and algorithms. In the current experiment a group of healthy adults (*n* = 14) was trained by professional trainers to up-regulate their beta1 (15–22 Hz) band for eight sessions. The control group (*n* = 18) underwent the same training regime but without rewards for increasing beta. In half of the participants trained to up-regulate beta1 band (*n* = 7) a systematic increase in tonic EMG activity was identified offline, implying that muscle activity became a foundation for reinforcement in the trainings. The remaining participants did not present any specific increase of the trained beta1 band amplitude. The training was perceived effective by both trainers and the trainees in all groups. These results indicate the necessity of proper control of muscle activity as a requirement for the genuine EEG-NFB training, especially in protocols that do not aim at the participants’ relaxation. The specificity of the information fed back to the participants should be of highest interest to all therapists and researchers, as it might irreversibly alter the results of the training.

## Introduction

In the last two decades, EEG-based neurofeedback (EEG-NFB) received vast popularity in clinical and paramedical practice, even though the therapeutic usage of this method was precariously ahead of the careful, systematic examination of its physiological mechanisms, confounding factors and possible side effects.

The method belongs to a broader category of biofeedback techniques aimed at altering various physiological parameters such as heart rate (ECG-feedback), muscle tension (EMG-feedback) and others. The trainings are based on the assumption that one can learn to change her/his brain physiological activity in a chosen oscillatory frequency by virtue of continuous feedback about its amplitude. On the contrary to research on brain computer interfaces (BCI) which concentrates on finding easily detectable and modifiable signals that can be reliably used by machine control algorithms, the EEG-NFB training is directed toward lasting changes of brain activity and behavioral improvement. The trainings aim to induce systematic increases/decreases of predefined specific EEG frequencies reflecting particular cognitive or behavioral functions.

The EEG-NFB has been tested as a treatment in a vast domain of neurological and psychiatric disorders, e.g., epilepsy (Sterman and Friar, 1972; Kotchoubey et al., 2001), attention deficit hyperactivity disorder (ADHD; Kaiser and Othmer, 2000; Fuchs et al., 2003; Kropotov et al., 2007), schizophrenia (Gruzelier et al., 1999; Surmeli et al., 2012) and even in traumatic brain injury (TBI) and stroke rehabilitation (for the review of clinical applications, see Yucha and Montgomery, 2008). In healthy subjects the EEG-NFB has been applied expecting behavioral and/or cognitive improvements (Arns et al., 2008; Reiner et al., 2014) and as a supportive training of cognitive performance in the elderly (Becerra et al., 2012; Staufenbiel et al., 2014).

All traditionally discriminated EEG bands have been used as a feedback source, i.e., slow cortical potentials (<2 Hz; Birbaumer, 1999; Heinrich et al., 2004; Strehl et al., 2006; Leins et al., 2007), theta (4–7 Hz; Egner et al., 2002; Raymond et al., 2005; de Zambotti et al., 2012), alpha (8–12 Hz; Egner et al., 2002; Raymond et al., 2005; Zoefel et al., 2011; Gruzelier et al., 2014), lower and higher beta (12–30 Hz; Cannon et al., 2009; Egner and Gruzelier, 2001, 2004) and gamma (>30 Hz; Keizer et al., 2010a,b; Staufenbiel et al., 2014). This large diversity of protocols (i.e., sets of frequency bands used to up- or down-regulate their amplitudes or their ratios) resulted from a belief that each frequency range is related to some specific cognitive functions. Even though instances of such frequency-to-function mapping have been documented (Wróbel, 2000, 2014; Wang, 2010; Anguera et al., 2013), their complex interactions do not allow for simplifying generalizations and require further investigations. Among others, the beta band has been posited to be an attention carrier (Wróbel, 2000, 2014), with specific, local increases of amplitude during attentional tasks positively correlating with correct performance in animals and humans (Bekisz and Wróbel, 1993; Buschman and Miller, 2007; Wróbel et al., 2007; Kamiński et al., 2012; Gola et al., 2013). The up-regulation of this band has been applied in more complex EEG-NFB training protocols as a supplementary treatment in ADHD (e.g., Lévesque et al., 2006; Leins et al., 2007) and as skill enhancement for sportsmen and the elderly (e.g., Rostami et al., 2012; Staufenbiel et al., 2014).

Research focusing on beta and gamma bands confronts the problem of EEG contamination by muscle activity. Electromyographic activity recorded on the surface of the skin is composed of high frequencies with most of the power concentrating between 20 Hz and 150 Hz (Criswell, 2011). In consequence, muscles located on the head (e.g., temporal, occipitofrontal and auricular muscles) or even more distally can interfere with the EEG, sometimes constituting a majority of the power in the higher frequencies (Goncharova et al., 2003; Whitham et al., 2007) and influencing most of the electrodes on the scalp (Goncharova et al., 2003; Yilmaz et al., 2014). The relation between these signals is further complicated by the fact that facial EMG was shown to be sensitive to numerous cognitive and affective processes, including cognitive load (Waterink and van Boxtel, 1994; Whitham et al., 2008). Muscle interference has been widely discussed in the context of EEG and EMG data analysis (for review see McMenamin et al., 2011; Muthukumaraswamy, 2013) but is not sufficiently recognized and controlled in EEG-NFB trainings (see “Discussion” Section and Enriquez-Geppert et al., 2017). The EEG signal analyzed offline can be iteratively examined and cleaned of any obscuring components. However, in the EEG-NFB the signal is analyzed online and transformed into stimuli immediately fed back to a trained person. All undetected artifacts modify the feedback signal. This might cause different effects in the case of various training protocols and therefore needs to be carefully considered while designing training protocols and algorithms.

Here we report a particular instance of this problem concerning the NFB up-regulation of the beta band. Since there is vast research on clinical and normal population reporting no control or one with doubtful effectiveness (e.g., Leins et al., 2007; Gevensleben et al., 2009a,b; Keizer et al., 2010a,b; Logemann et al., 2010; Meisel et al., 2014; see “Discussion” Section), we conducted an experiment to assess the possible impact of muscle activity on EEG-NFB results. We applied the beta up-regulation set-up, commonly used as training aimed at improving attention (Egner and Gruzelier, 2001; Vernon et al., 2003; Egner et al., 2004; Logemann et al., 2010; Ghaziri et al., 2013). Healthy, young participants were trained to voluntarily increase the amplitude of beta1 band oscillations (15–22 Hz) recorded from the leads overlying the areas of the frontoparietal attention network. Examination of the raw signal revealed in a subgroup of participants a substantial muscle employment which increased systematically in the course of the session, mimicking the expected increase in the beta1 band. If the muscle related effects had gone unnoticed, the conclusion of our study would have been falsely positive stating a successful upregulation of the beta1 band. We discuss the need for proper muscle control for a reliable NFB training in the light of the current EEG-NFB literature.

## Materials and Methods

### Participants

Thirty-two male healthy university students, age *m* = 21.97 ± 1.88 years (mean ± standard deviation), were recruited for the experiment. The experiments were approved by the local ethics committee (Bioethical Committee at the Military Institute of Hygiene and Epidemiology). All subjects were informed about the study and gave their written informed consent for participation in the experiment in accordance with the Declaration of Helsinki.

### The EEG-Neurofeedback Training

The subjects were randomly assigned to one of the three training groups: beta plus (B+), aimed at increasing the amplitude of beta1 (15–22 Hz) oscillations (*n* = 14), beta minus (B−) dedicated to down-regulation of the beta1 oscillatory activity (*n* = 6) and sham (SH) group, receiving pseudo-feedback (generated by a computer algorithm), unrelated to the brain’s EEG signals (*n* = 12). The participants were unaware of their group affiliation and uninformed about the existence of the sham group to prevent loss of motivation.

The training sessions were performed using a customized version of the commercial EEG DigiTrack Biofeedback system (ELMIKO MEDICAL Sp. z o. o.). Each participant had a personal code, which was recognized by the program and started group-dependent feedback protocol. At the user (trainer) level, the program displayed set-ups only for two groups: beta plus and beta minus. Sham protocols were run under facade of these set-ups (half as B+ and the other half as B−). The trainings were conducted by hired professional NFB trainers. In order to reduce possible nonspecific effects trainers were instructed not to additionally motivate the trainees. Over a period of 1–2 months the subjects underwent eight training sessions (one to two trainings per week). During the session subjects were seated in a chair in front of a 17″ computer LCD screen (~70 cm from the screen). Each session consisted of 10 blocks of 3 min duration each. The session started after mounting the EEG electrodes with a short (*ca.* 2 min) resting period in order to accustom the participants with the training situations and screen the control sample of the EEG signal.

EEG was recorded from F3, F4, P3 and P4 sites in 10–20 standard, with linked ears as a reference and ground electrode placed at the Pz. Thus, the electrodes were positioned over the frontoparietal attention network nodes (Gross et al., 2004; Donner et al., 2007; Siegel et al., 2008). The signal was sampled at 250 Hz and band-pass filtered between 0.16 Hz and 70 Hz, with a notch filter at 50 Hz. A fast Fourier transform (FFT) spectrogram was computed for each electrode. The feedback parameter was obtained by averaging the FFT amplitudes over the beta1 range and across the electrodes. The FFT window of 2.07 s (512 point, giving 0.49 Hz resolution) was sliding with 92% or 77% overlap and, accordingly, the amplitude values presented to the trainer and used for feedback were updated with 200 or 500 ms delay. Windows overlap and the feedback delay varied between subjects (due to two software versions used in the study) but were constant for each person and randomly distributed among the experimental groups. There was no difference in results of participants trained with these two settings.

**Figure 1:**
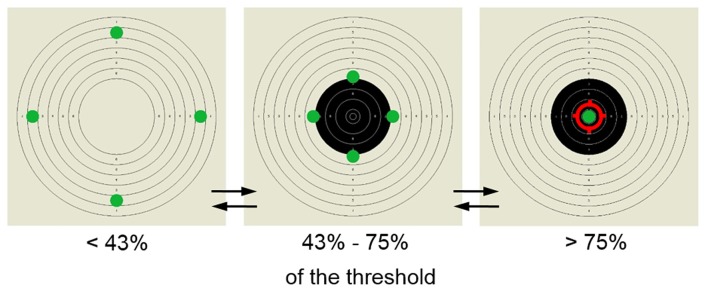
**Screen-stimulus used for the neurofeedback (NFB) training.** Participants’ goal was to move the four dots initially located on the outer edges of the shooting target to its center. The reward was provided in two steps—when the amplitude of trained band reached over 43% of the pre-set threshold the shooting target was filled in with black central rings, when it exceeded 75% the dots met in the center and the shooting aim appeared as a sign of successful performance.

The training display consisted of a shooting target presented in the background and four green dots moving inwards and outwards along vertical and horizontal axes (Figure 1). The feedback information about the amplitude of beta1, was provided by the synchronized movement of the dots in B+ and B− groups. In the sham group a predefined algorithm controlled the movement of the dots. When the amplitude changed in the intended direction (towards the threshold value set manually by the trainers) all dots moved inwards. The subject’s goal was to make the green dots meet in the center. To boost the participants’ motivation and to make the training more involving additional reinforcements were provided. When the beta1 amplitude reached 43% of the threshold, the display was complemented with black rings within the high-scored area of the shooting target. When the beta1 amplitude reached 75% of the threshold value, a red ring in the center of the shooting target was presented, signaling achievement of the goal. The threshold defining the required value of the beta1 band amplitude was adjusted manually by the trainers during the session to provide a relatively constant rate of reward, thus encouraging the subjects to continuously improve their performance.

### Processing of the EEG Data

Raw EEG data were exported from the DigiTrack environment to the European Data Format (EDF) and further analyzed using the EEGLAB software (Delorme and Makeig, 2004) and self-written MATLAB scripts. The off-line analysis involved similar preprocessing of the data as the online feedback computation, to obtain the same frequency bands to those produced by the EEG-NFB apparatus. The continuous data recorded during training session were mean corrected and filtered to remove frequencies lower than 0.5 Hz and higher than 70 Hz. Notch filtering was applied at 50 Hz. The signal was split into 1 s epochs, which in turn were searched for artifacts with the EEGLAB function *pop_autorej*. An epoch was rejected from all the channels if any data point in this epoch exceeded 5 standard deviations from the amplitude of the signal on any of the channels. The algorithm proceeded iteratively—if the number of epochs classified for exclusion exceeded 5% of the data, the procedure was repeated with a more liberal exclusion criterion (increased by 0.5 standard deviation). Furthermore, to remove muscle artifacts, for every subject we removed whole training blocks, in which higher frequencies (22–45 Hz, referred to in DigiTrack software as beta2 band) diverged by more than 3 standard deviations from the individual mean of that participant. The procedure ran iteratively until no such cases were found. In effect, 3.05% of the data was removed, including three full sessions (belonging to two subjects). The FFT analysis was computed separately for each 3 min training block with a sliding Hanning window 512 points-long with a 92% overlap.

**Figure 2:**
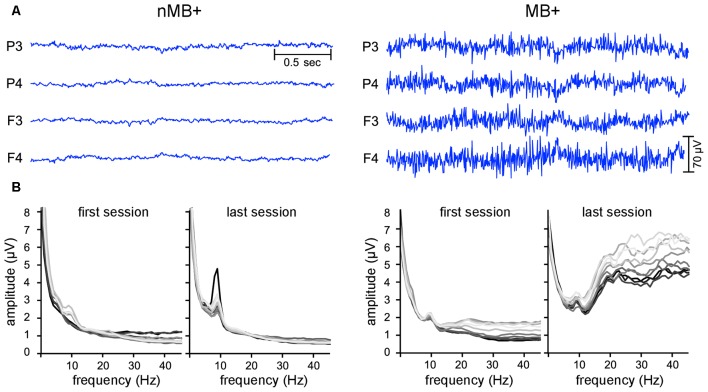
**Examples of the EEG data with and without the EMG contamination. (A)** Raw EEG signal from four electrodes used for training. **(B)** Frequency spectra (fast Fourier transform (FFT)) averaged for all four NFB electrodes. Each line corresponds to a one 3-min block in the session, with each consecutive block marked with a brighter color (the darkest line—the first block, the lightest one—the tenth block). On the left: data from an exemplar subject with the EEG spectrum undistorted in the high frequency range (nMB+). On the right: data from a subject with increased amplitudes at high frequency part of the spectrum identified as originating from muscle activity (MB+).

The visual inspection of the raw signal and the FFT spectra (performed after all cleaning steps described above) revealed, that the signals of some participants were dominated by frequencies above 15 Hz (Figure 2). Since such oscillations constituted the majority of some participants’ signal, cleaning algorithms relaying on signal distribution parameters e.g., mean and standard deviation were unable to detect and reject them during offline automatic data processing. These signals were characterized by long sweeps of sharp high frequency oscillations characteristic for EMG (Criswell, 2011). In these cases, a typical logarithmic-like shape of the FFT curve was substantially distorted by an elevation spanning from higher to lower parts of the spectrum.

Therefore, we asked three independent judges to visually inspect the raw signals and the FFT spectra from all blocks and sessions of each participant and to classify the sessions as contaminated by muscle activity if FFT spectrum was distorted above 15 Hz in the majority of blocks (Figure 2; Criswell, 2011). The judges listed the participants who exhibited such a pattern in more than half of their sessions as muscle employing ones.

To confirm that it is possible to distinguish muscle-employing participants from the whole sample in an automated approach we applied two additional methods of classification: k-means clustering and logistic regression. For both these methods we used solely the amplitudes of beta2 as the bases of classification.

When applying the k-means method we asked for two clusters, to prove that our division into muscle employers and others is the most prevalent pattern in the data. The analysis was performed with the use of beta2 amplitudes from all available recordings (10 blocks × 8 sessions for each subject, 3.05% of blocks were missing, due to previous data cleaning and were substituted with the average of a given participant). The algorithm was set to minimize absolute deviations within clusters by calculating the median along predefined dimensions (Manhattan distance).

The logistic regression was applied as a supervised method of data classification. The binary classifier (muscle-employing/other) was guided by the classification made by independent judges. The mean beta2 amplitude from all available recordings for each participant constituted the predictor value. The regression line, fitted to the data, quantified the relationship between beta2 amplitude and the probability of belonging to one of the two categories. The decision criterion was established at a probability of 0.5.

In addition to the trained beta1 band (15–22 Hz), we reported on the alpha (8–12 Hz) and beta2 (22–45 Hz) flanking bands, as they are capable of showing potential specificity of the training effects.

### Statistical Analyses

The amplitude values in each frequency band were averaged from the four electrodes (F3, F4, P3, P4) to reproduce the training setup averaging online the amplitudes from all channels. We confirmed with a three-way ANOVA of group, session and electrode that there were no significant differences between individual electrodes with respect to amplitudes of analyzed bands (no significant effects of electrode or interactions including this factor, all *p* > 0.201).

The main goal of the analysis was to compare the effects of the training in the participants employing and not employing muscles in their performance. The most prominent characteristic of the muscle-employing subjects was a pronounced elevation of high frequency amplitudes and their high variability among different blocks. In order to maintain the relations between individual subjects, as present in the raw data, we chose to perform a between-subject standardization (by subtracting the mean and dividing by standard deviation from all blocks/sessions across all participants). This procedure performed for each band separately enables a direct comparison of different frequency bands as it shifts the values to a common range (*z* space). Additionally, to ensure that the observed effects, even if different in absolute size, are common for the group and not driven by single cases we repeated our analysis with the within-subject *z*-scores (using individual mean and deviation for each subject).

We verified the effects of the EEG-NFB during the course of the session (the within session effects) and in consecutive sessions (the between session effects). For the within session effects, the values for each of the 10 blocks were obtained by averaging across all the sessions. For the between-session effects, the values were obtained by averaging all the blocks constituting each session. The missing session averages (only 3 per all 256 data points in all participants) were substituted with mean values interpolated from the directly preceding and following sessions.

Considering the small number of participants assigned to the B− protocol for the sake of further analyses we decided to combine it with the sham group to create a single control condition, further referred to as control group (CON). Before combining the groups we confirmed with three-way ANOVA of group, session and band that there were no significant differences between these groups (no significant effect of group or interactions including this factor, all *p* > 0.208). Since seven participants identified to have a steady EMG contamination belonged to the B+ protocol (50% of this group), we split this group into MB+ (muscle-employing participants from B+ group, *n* = 7) and nMB+ (participants not employing muscles from B+ group, *n* = 7). Their performance was compared to the results of the control group (CON, *n* = 18). The three-way ANOVAs were computed for within and between session effects with “time” (blocks 1–10 or sessions 1–8), “band” (alpha, beta1, beta2) as within subject factors and training “group” (MB+, nMB+, CON) as between subject factors. The Greenhouse-Geisser correction (G-G) was applied when the data did not meet the sphericity. We considered the results to be significant when the *p* value was below 0.05. For significant interactions *post hoc* pairwise comparisons were provided. For clarity of the presentation, from multiple pairwise comparisons between consecutive time points, we show the comparison of the first and the last blocks/sessions and prove the gradual character of the change by fitting a linear trend.

### Self-Reports

After completing the training the participants were asked to assess: (1) the effectiveness of the NFB training; (2) the influence of the training on their functioning outside the sessions; (3) their ability to evoke the state from the trainings outside the sessions; (4) their progress in the ability to control visual stimulus during the trainings; and (5) their implemented strategies (if any).

## Results

### Electroencephalographic Data

Fourteen healthy subjects took part in eight sessions of the EEG-NFB training that aimed to up-regulate the beta1 band amplitude. Another 18 participants who underwent the same training regime but were not rewarded for increasing beta1 amplitude formed the control group to account for the unspecific training factors.

To assess the possible impact of muscle activity on the EEG-NFB results we divided the participants into subgroups based on the presence of the extended muscle contamination in their EEG signal. To assure the validity and consistency of the ensuing division three different classification methods were used. Based on the screening of the raw signal and the shape of the FFT spectra competent judges marked nine subjects as muscle-employing. Their assessments agreed in 91% of cases (eight participants were identified by all three judges, and one by two judges).

**Figure 3:**
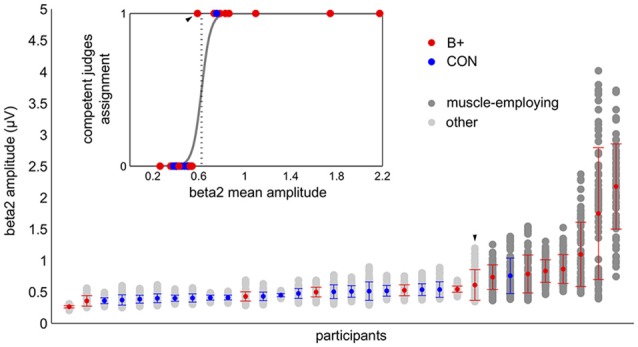
**Automatic classification of the participants based on beta2 amplitude: k-means (main graph) and logistic regression (insert).** In the main graph the light and the dark gray dots correspond to the values of the beta2 amplitude from the single training blocks. The vertical bundles of dots display all of the training data of the individual participants, which constituted the basis for k-means clustering. The graph summarizes the results of the k-means classification: dark gray dots—muscle employing participants, light gray dots—others. The colored dots (in both graphs) correspond to the grand average beta2 of the person with red indicating the participants from the beta up-training group (B+) and blue those from the control group (CON). The participants are sorted by the increasing average of their beta2 amplitude. The error bars represent standard deviations. The embedded graph shows the result of a logistic regression analysis supervised by competent judges group assignments (code 1—muscle employing participants, code 0—others). The dotted vertical line marks the decision criterion. The participants whose grand mean exceeded this value were classified as muscle employing. Both automatic methods provided concurring results. Note the ambiguous case which was classified by competent judges as muscle employing but did not reach the decision criterion in both automatic classifications (marked on the graph with the arrow head).

K-means clustering with assumed two clusters divided the data into groups of eight and 24 participants. Eight out of nine subjects marked as muscle-employing during the visual inspection were classified as such by the k-means algorithm (see Figure 3). All 23 participants judged not to employ muscles were in the second cluster. The same result was obtained with logistic regression (Figure 3). The increase in beta2 amplitude significantly raised the probability of a subject being classified as muscle employing (*β* = 25.92;* t* = 3.87; *p* < 0.001). It is worth noticing that while visual inspection was based on raw signals as well as on the shape of entire FFT spectrum, automatic classifications, relying exclusively on the amplitude of beta2 band lead to very similar results, proving that changes in the high frequencies are the critical feature distinguishing these two groups. All approaches resulted in the same outcome in 31/32 cases. In the single ambiguous case we leaned toward the automatic classification and included this subject in the control group.

**Figure 4:**
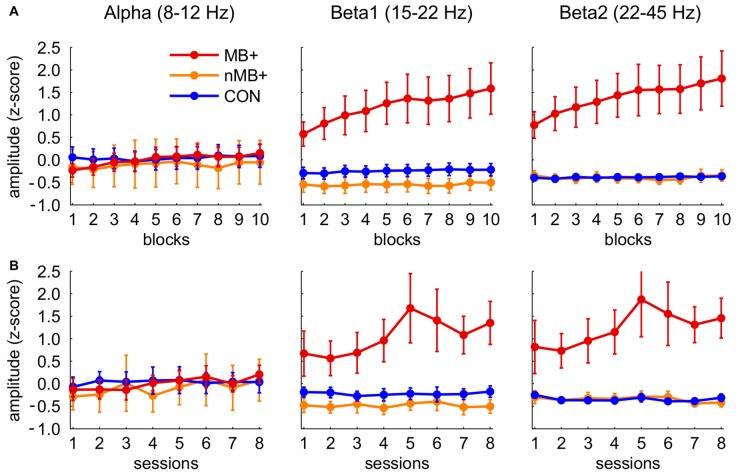
**Standardized (between subjects, see “Materials and Methods” Section) amplitudes of the three frequency bands in the three training groups (MB+, *n* = 7; nMB+, *n* = 7; CON, *n* = 18; for statistical significance see “Results” Section). (A)** Consecutive 10 blocks averaged across sessions. **(B)** Means of eight consecutive sessions. Error bars represent standard error of the mean.

We compared the effects of the training on the muscle employing participants trained to upregulate the beta band with other groups. The analysis of the within session effects (Figure 4A) revealed a significant three-way interaction of block, band and group factors (*F*_(36,522)_ = 4.23, *p* = 0.001, *η*^2^ = 0.226). The statistics for the main and the interaction effects are shown in Table 1. We observed a general increase of amplitudes in MB+ during training session, with the magnitude varying between the bands. The most pronounced increase was in beta1 (first block: *m* = 0.58, last block: *m* = 1.59, *p* < 0.001, linear trend at *p* = 0.029) and beta2 (first block: *m* = 0.77, last block: *m* = 1.81, *p* < 0.001, linear trend at *p* = 0.029). In both of these bands the amplitudes during entire training session were significantly higher in this group than in nMB+ or CON (all *p* < 0.001). A smaller, yet significant increase was also present in the alpha band (first block: *m* = −0.23, last block: *m* = 0.15, *p* = 0.001, linear trend at *p* = 0.018). The mean alpha amplitude did not significantly differ between groups (all *p* > 0.745). MB+ was the only group that exhibited any amplitude changes during the training session. All comparisons between the first and the last blocks, for all bands in the remaining groups turned out insignificant (all *p* > 0.361).

**Table 1:** **Results of a three-way ANOVA with time, band and group factors**.

	Within sessions				Between sessions			
	*F*	*df1*	*df2*	*p*	*F*	*df1*	*df2*	*p*
Time	13.79	9	261	0.000	2.13	7	203	0.112
Band	0.90	2	58	0.368	0.82	2	58	0.391
Group	7.35	2	29	0.003	5.605	2	29	0.009
Time × Band	2.88	18	522	0.041	0.91	14	406	0.453
Time × Group	8.58	18	261	0.000	1.45	14	203	0.215
Band × Group	6.70	4	58	0.002	6.06	4	58	0.004
Time × Band × Group	4.23	36	522	0.001	1.39	28	406	0.215

In the analogous analysis performed for the between session effects (Figure 4B) the three-way interaction of session, band and group factors appeared insignificant (*F*_(28,406)_ = 1.39, *p* = 0.215, *η*^2^ = 0.087), demonstrating a lack of systematic changes in the EEG amplitudes across sessions similar to those observed within sessions. However, beta1 and beta2 amplitudes were significantly higher across sessions (all *p* < 0.001) in MB+ (beta1 *m* = 1.05 ± 1.09; beta2 *m* = 1.23 ± 1.152) than in nMB+ (beta1 *m* = −0.48 ± 0.40; beta2 *m* = −0.35 ± 0.25) and CON (beta1 *m* = −0.53 ± 0.50; beta2 *m* = −0.40 ± 0.21), as shown in the significant interaction of band and group factor (*F*_(4,58)_ = 6.06, *p* = 0.004, *η*^2^ = 0.295). There was no difference between groups in the alpha band amplitude (all *p* > 0.764).

The same analyses performed on the *z*-scores computed for each participant separately confirmed the effects previously observed in the beta1 and beta2 bands. The amplitude of these two bands increased within session in the MB+ group and did not change significantly in the other two groups as revealed by the interaction effect (*F*_(18,261)_ = 2.38, *p* = 0.032, *η*^2^ = 0.141). The increase of the alpha amplitude visible in the previous approach appeared to be equal to the one observed in higher frequencies after its normalization to the subject-specific variability range. This is evidenced by the lack of significant differences between the bands in the MB+ group (interaction of band and group for the within session: *F*_(4,58)_ = 0.27, *p* = 0.827, *η*^2^ = 0.018 ; between session: *F*_(4,58)_ = 0.31, *p* = 0.744, *η*^2^ = 0.021).

The impact of muscle activity on the EEG spectrum increased with frequency. However, the training related increase in amplitude was proportional to the amount of muscle contamination in the particular frequency. The results showed that after separating muscle-employing participants from the group trained to increase beta1 band we were unable to observe any effects of the NFB training as indicated by no significant differences between the nMB+ and control group participants.

### Self-Reports

The participants expressed their opinion about the EEG-NFB trainings on a Likert scale (1—not effective, 5—effective). The assessment of the effectiveness of the training varied between groups (*F*_(2,29)_ = 5.65, *p* = 0.008, *η*^2^ = 0.280). Subjects assigned to the control group expressed a more positive opinion about the effectiveness of the training (*m* = 4.17 ± 0.71) than those from the MB+ (*m* = 3.29 ± 0.76) and nMB+ (*m* = 3.00 ± 1.29). Consistently, the majority of subjects from the CON group (13 out of 18) declared that the trainings had positive influence on their functioning outside the sessions, the same was true only for two out of seven subjects in the MB+ and only two subjects in the nMB+ group ($\chi_{\left(2,32\right)}^{2}$ = 6.03, *p* = 0.049). About half of all the participants declared their ability to transfer the state maintained during the sessions onto other situations (half of the nMB+, two thirds of the CON and only one person form the MB+ group, $\chi_{\left(2,31\right)}^{2}$ = 5.55, *p* = 0.063).

There were no differences between the groups in the reported ability to control the visual stimulus (*F*_(2,29)_ = 2.26, *p* = 0.122, *η*^2^ = 0.135). All groups declared that their ability to control the visual stimulus increased during the trainings (CON: *m* = 4.33 ± 0.97, MB+: *m* = 4.14 ± 0.69, nMB+: *m* = 3.43 ± 1.13). During the trainings the participants were left without any specific instruction. In post trainings self-reports, we asked them if they implemented any strategies to control the visual stimulus. In all groups the majority of subjects declared to apply some strategies during the NFB sessions ($\chi_{\left(2,32\right)}^{2}$ = 0.169, *p* = 0.919). The participants indicated strategies such as: (1) looking at one point; (2) solving logical puzzles; (3) visualizing places; (4) relaxing; (5) focusing and calming down; and (6) singing songs in their minds. Four subjects from the MB+ group and one from nMB+ mentioned in their reports changing the muscle tension. Therefore, about half of the participants from MB+ were aware of the possibility to use muscle tension to control the feedback and half were not. However, these numbers are too small for direct statistical comparisons. The self-aware subjects had a slightly better opinion about the effectiveness of the training and perceived ability to control the visual stimulus than MB+ who were unaware of their muscle employment (effectiveness *m* = 3.00 ± 0.816 vs. *m* = 3.67 ± 0.58, perceived control *m* = 3.75 ± 0.50 vs. *m* = 4.67 ± 0.58).

## Discussion

In the present experiment performed on healthy adults, we failed to observe a change of the EEG activity in the trained beta1 (15–22 Hz) band despite the positive reception of the trainings effects reported by both the trainers and the trainees. However, we observed extensive muscle employment, which increased during the training sessions. We argue that in the reported experiment, in the subgroup of participants the EEG-NFB training was taken over by the EMG signal, which became the foundation for incentive-based learning. This conclusion is supported by reports showing that EMG is more susceptible to feedback modification than EEG (DeGood and Chisholm, 1977; Maurizio et al., 2013). Indeed, all but one participants who increased muscle activity belonged to the training group up-regulating the beta1 band. This group was rewarded for amplifying amplitude of beta1, which is at the lower edge of the EMG spectrum (Criswell, 2011). On the contrary, such excessive artifacts were not present in participants trained to down-regulate beta1 activity and present only in 1 out of 12 subjects trained in the sham protocol. In that participant the muscle activity did not change systematically in the course of the training.

We cannot eliminate the possibility that in the beta1 activity recorded from the participants identified as the muscle employing group (MB+) there was also a contribution of a neuronal origin overshadowed by muscular activity. However, in the analysis restricted to the subjects who did not employ muscles for trainings, no modifications in the beta1 range were detected, suggesting ineffectiveness of the performed EEG-NFB training. The susceptibility of the beta1 band for the NFB training has yet to be established with larger sample sizes.

Subjects generally declared that their ability to control the visual stimulus increased during the course of the trainings. Surprisingly, the participants from the control group (who did not show any changes in the EEG during the trainings) were most positive about the training effects. This counter-intuitive result may point out the presence of a placebo effect in the NFB trainings. It acted as if the effect was most pronounced when undisturbed by real control of the ongoing feedback (the case of MB+, which was less pleased with the effectiveness of the NFB). A majority of participants reported using various strategies during the trainings. The employment of muscle tension to control the visual feedback stimulus was reported post-training by four participants from MB+, the remaining subjects were unaware of using this strategy or failed to mention it in their reports. At the beginning of the experiment the participants were informed about the basic mechanisms of the EEG-NFB e.g., its relation to the ongoing brain activity. To assure a high quality of the recorded signals they were asked to sit still, and trainers intervened whenever they noticed excessive movement or other type of undesirable behavior. The fact that half of subjects trained to up-regulate the beta1 band managed to increase their muscle activity (in half of the cases unwittingly) shows that such behavior cannot be efficiently controlled and eliminated by trainers alone.

Our experiment strongly supports the need for effective automatic on-line control of muscle activity during the EEG-NFB trainings, in particular in the protocols aiming to up-regulate higher frequency bands. Proper muscle control is a requirement not solely to acquire a high quality EEG signal but primarily to accomplish a genuine EEG-NFB training. Muscle control must be recognized as standard part of the NFB procedure, to successfully prevent participants from unintentionally using muscle tension to change the signals registered by the EEG electrodes during the training. Various mathematical algorithms were proposed and validated as effective in removing the EMG components from the EEG signal (McMenamin et al., 2011; Fitzgibbon et al., 2016), however, most of them require high density multichannel recordings and as such are not applicable in the case of typical NFB setups. It was also proposed to model, fit and subtract individual EMG spikes from the EEG channels (Nottage et al., 2013)—such an approach does not require multiple recording channels but needs powerful computers and sophisticated software.

The strategy suggested in NFB guidebooks (Demos, 2005) is based on the intervention of the trainer, who is expected to instruct the trainee to relax, adopt a proper, comfortable position and to avoid muscles contraction. Trainers are expected to visually screen the recorded EEG and FFT spectra to detect sweeps of EMG activity and to instruct the trainees to correct their behavior. While this is the demanded minimum that can be done without adequate hardware and software support, present experiment shows that such a strategy may be inefficient at least in the trainings aiming to up-regulate high frequencies. Surprisingly, the reports provided by the authors in a clinical research concerning mostly the NFB applications for ADHD treatment in children do not refer to the issue of muscle control (Lévesque et al., 2006; Leins et al., 2007; for review see Lofthouse et al., 2012). It remains widely neglected also in the research conducted on healthy participants in which high frequency (beta and gamma) training is used in e.g., for cognitive improvement (Keizer et al., 2010a,b; Logemann et al., 2010). The articles presenting various control procedures are noticeable exceptions (e.g., Bird et al., 1978; Berner et al., 2006; Hoedlmoser et al., 2008; Kober et al., 2013; Witte et al., 2013). Based on this research three main approaches to control muscle activity can be distinguished.

Probably the most common, although only incidentally described as serving for artifact control, is the usage of multiband protocols aiming at simultaneous manipulation of two or more bands, or maximization of their proportion (e.g., up-regulating SMR while simultaneously decreasing theta and beta2). Rarely was such protocol claimed as serving for the eye blink (theta) and muscle (beta2) control (Kober et al., 2013; Witte et al., 2013). Even though such an approach can be effective in controlling muscle-related activity it is threatened by physiological invalidity when the contrasted bands are directly flanking (Ros et al., 2013). It is highly plausible that (at least) the edges of the neighboring bands are mutually interdependent and if left unconstrained, they tend to change their amplitude in the same direction. The interdependence of the flanking frequency bands (on the example of theta, alpha and beta) was demonstrated as moderate to strong (by means of correlation of the within-subject changes during training, 0.5 < *r* < 0.7 (Ros et al., 2013). Thus, implementation of such protocols, although effective for the control of muscle artifacts, might lower the effectiveness of training.

Our study shows that the classification based solely on the beta2 amplitude proved that the changes in this frequency are a feature distinguishing the subjects who train based on the EMG activity from others. Thus, controlling this single parameter is sufficient to assure good quality of the feedback information. It can be implemented as an amplitude threshold on one of the high frequency bands, as the signal of the muscle origin have an amplitude far bigger than the neuronal one (procedure employed i.a. by Hoedlmoser et al., 2008). When the signal exceeds this threshold the participants are left without a reward and the training is interrupted. This method can be easily implemented in commercial software, but it does not completely exclude the risk of under threshold manipulation of muscle tension. Many of the articles that implement these solutions, report the use of an amplitude threshold in the range of 100–120 μV (e.g., Gevensleben et al., 2009a,b; Meisel et al., 2014). Our data clearly shows that it is perfectly feasible to gain control over the NFB apparatus with muscle activity in the range of 70 μV or lower (Figure 2). Since different apparatus and online signal processing settings influence the range of the recorded EEG values the amplitude threshold should not be generic but separately calibrated.

Finally, some authors used additional EMG recordings of facial muscles (Berner et al., 2006), neck-muscles (Bird et al., 1978) or the chest belt measuring the chest wall movements (Berner et al., 2006). In these experiments positive feedback was conditional upon ongoing EMG activity and provided only if the latter did not exceed established threshold. As this method unambiguously distinguishes between neuronal and muscular signals, it is the most precise one but also the most demanding in implementation (requiring additional hardware and software facilities and prolonging the preparation time).

Muscle activity can differently influence various training protocols, so it should be specifically approached with regard to the training band and behavioral goal. In the case of the protocols aiming to up-regulate high frequency bands, the EMG signal can result from a head or upper body muscle strain. It is especially plausible in trainings focusing on attention (which constitute many of high frequency protocols, e.g., Egner and Gruzelier, 2004; Cannon et al., 2006, 2009), as they do not instruct subjects to relax during the trainings. The situation is different for the NFB trainings aiming at subjects’ relaxation, most often related to alpha protocols (e.g., Egner et al., 2002; van Boxtel et al., 2012). Interestingly, few articles directly comparing the EEG and EMG feedback employed the alpha band and have shown that down-regulating the amplitude of the muscle signal in the EMG-feedback can be equally or even more effective in increasing the alpha amplitude when compared to the direct alpha training (DeGood and Chisholm, 1977; Moore et al., 2000). Both these procedures resulted in subjects’ relaxation as supported by physiological indices such as heart and respiratory rates (DeGood and Chisholm, 1977). In this comparison, the EMG training was explicitly described by participants as easier in the post training survey. Thus, the EMG-feedback can be considered as a valid replacement of the EEG-NFB protocols aiming at the alpha band up-regulation.

We conclude that the activity from the EEG electrodes might be overwhelmed by the stronger and easier to control EMG signals, which in turn becomes a foundation for the feedback reinforcement. This might cause different effects in various training protocols and therefore needs to be carefully considered while designing training protocols and algorithms. Online data analysis and quality of information fed back to the participants should be of the highest interest to all therapists and researchers, as any shortcomings at this stage irreversibly alter the training and cannot be fixed in further offline data processing. Extensive effort should be undertaken within the NFB community to develop, validate and implement in experimental and commercial setups efficient automatic artifact detection algorithms.

## Author Contributions

KP and KJ: data analyses, main conclusions, article drafting (equal contribution). JR: interpretation of the results, proofreading. RK and MS: data collection. MM: experimental setup, data collection. AW and EK: verification of main concept and conclusions, proofreading.

## Conflict of Interest Statement

The authors declare that the research was conducted in the absence of any commercial or financial relationships that could be construed as a potential conflict of interest.
